# Advancements in autologous peripheral nerve transplantation care: a review of strategies and practices to facilitate recovery

**DOI:** 10.3389/fneur.2024.1330224

**Published:** 2024-03-08

**Authors:** Guoying Xu, Xiaodi Zou, Yanzhao Dong, Ahmad Alhaskawi, Haiying Zhou, Sohaib Hasan Abdullah Ezzi, Vishnu Goutham Kota, Mohamed Hasan Abdulla Hasan Abdulla, Olga Alenikova, Sahar Ahmed Abdalbary, Hui Lu

**Affiliations:** ^1^Operating Theater, Shaoxing City Keqiao District Hospital of Traditional Chinese Medicine, Shaoxing, Zhejiang, China; ^2^Department of Orthopedics, The Second Affiliated Hospital of Zhejiang Chinese Medical University, Hangzhou, China; ^3^Department of Orthopedics, The First Affiliated Hospital, Zhejiang University, Hangzhou, Zhejiang, China; ^4^Department of Orthopedics, Third Xiangya Hospital, Central South University, Changsha, Hunan, China; ^5^School of Medicine, Zhejiang University, Hangzhou, Zhejiang, China; ^6^Department of Neurology, Republican Research and Clinical Center of Neurology and Neurosurgery, Minsk, Belarus; ^7^Department of Orthopedic Physical Therapy, Faculty of Physical Therapy, Nahda University in Beni Suef, Beni Suef, Egypt

**Keywords:** peripheral nerve injuries, autologous nerve transplantation, nursing care, functional impairments, rehabilitation

## Abstract

Autologous peripheral nerve transplantation, a pioneering technique in nerve injury treatment, has demonstrated remarkable progress. We examine recent nursing strategies and methodologies tailored to various anatomical sites, highlighting their role in postoperative recovery enhancement. Encompassing brachial plexus, upper limb, and lower limb nerve transplantation care, this discussion underscores the importance of personalized rehabilitation plans, interdisciplinary collaboration, and innovative approaches like nerve electrical stimulation and nerve growth factor therapy. Moreover, the exploration extends to effective complication management and prevention strategies, encompassing infection control and pain management. Ultimately, the review concludes by emphasizing the advances achieved in autologous peripheral nerve transplantation care, showcasing the potential to optimize postoperative recovery through tailored and advanced practices.

## Introduction

Peripheral nerve injuries (PNIs) pose a significant clinical challenge, often leading to severe functional impairments and disability (1, 2). Treatment options for peripheral nerve injuries (PNIs) vary based on the specific type and severity of the injury, including surgical repair, nerve transplantation, physical therapy, medication, and rehabilitation aids (3–5). Autologous nerve transplantation, also known as nerve grafting, is a surgical procedure that entails using a segment of nerve tissue to bridge the gap between the ends of a damaged nerve. While autologous nerve grafts are considered the gold standard due to their exceptional regenerative capacity, they are constrained by donor site length limitations and the potential for neuroma formation. Autologous nerve transplantation primarily finds application in repairing peripheral nerve injuries that leave gaps too extensive for simple nerve end suturing. Critical factors such as nerve parameters (e.g., location, length, and shape), donor nerve cross-sectional area, and its compatibility with the damaged nerves need careful consideration during the donor selection phase. Patient preferences also demand assessment since nerve harvesting may result in impairment, attenuation, or complete loss of function at the donor site in a significant number of cases. Consequently, autologous nerve transplantation remains the cornerstone for treating segmental nerve defects that are unsuitable for primary repair. In a study focusing on eight patients with complete high sciatic nerve injuries featuring extended defects (6) (>10 cm), a surgical intervention was performed involving autologous nerve grafting using the tibial nerve. Postoperative assessments conducted over a 36-to-60-month follow-up period included muscle strength and sensory function evaluations. Motor recovery was classified as “good” or “very good” (M3–M4) in 62.5% of the cases, with five out of the eight patients exhibiting such improvement. However, plantar flexion remained suboptimal in the remaining three patients. Sensory function was similarly encouraging, with “good” or “very good” (S2–S3) recovery noted in six patients, while two patients experienced “inadequate” (S4) sensory outcomes. Data on 4,331 patients who underwent reconstructive surgery for peripheral nerve abnormalities between 2015 and 2020 was gathered for a study (7). The results showed that after 2018, allograft utilization grew dramatically from 21.5 to 29.6%, while conduit utilization reduced from 60 to 54.7% and nerve autograft utilization dropped from 18.6 to 15.8%.

The rapid development of different materials as a substitute for nerve autografts in mending peripheral nerve defects has been facilitated by advancements in biomedical techniques. Research has focused on the use biomaterial-based nerve conduits for repairing the peripheral nerve defects.

Compared to autologous nerve grafting, nerve conduit repair of nerve defects eliminates the risk of donor site morbidity and achieves comparable results (8). However, its use is constrained by an optimal length, which may limit its applicability in certain scenarios. Studies found that regrowth and functionality were optimal for conduits of lengths ≤3 cm but deteriorated for lengths >3 cm (9).

In order to speed up nerve regeneration and bridge wide nerve gaps, supporting cells, such as stem cells or growth factors, have been added to the nerve conduits, which have attracted the greatest attention. Cell-based nerve conduits holds substantial promise in studies. However, significant challenges exist in its application in current and future clinical contexts. One is ensuring the safety of cell transplantation. Another obstacle is the extended waiting period required to prepare these autologous cell sources, which could potentially result in missing the critical treatment window.

**Figure 1:**
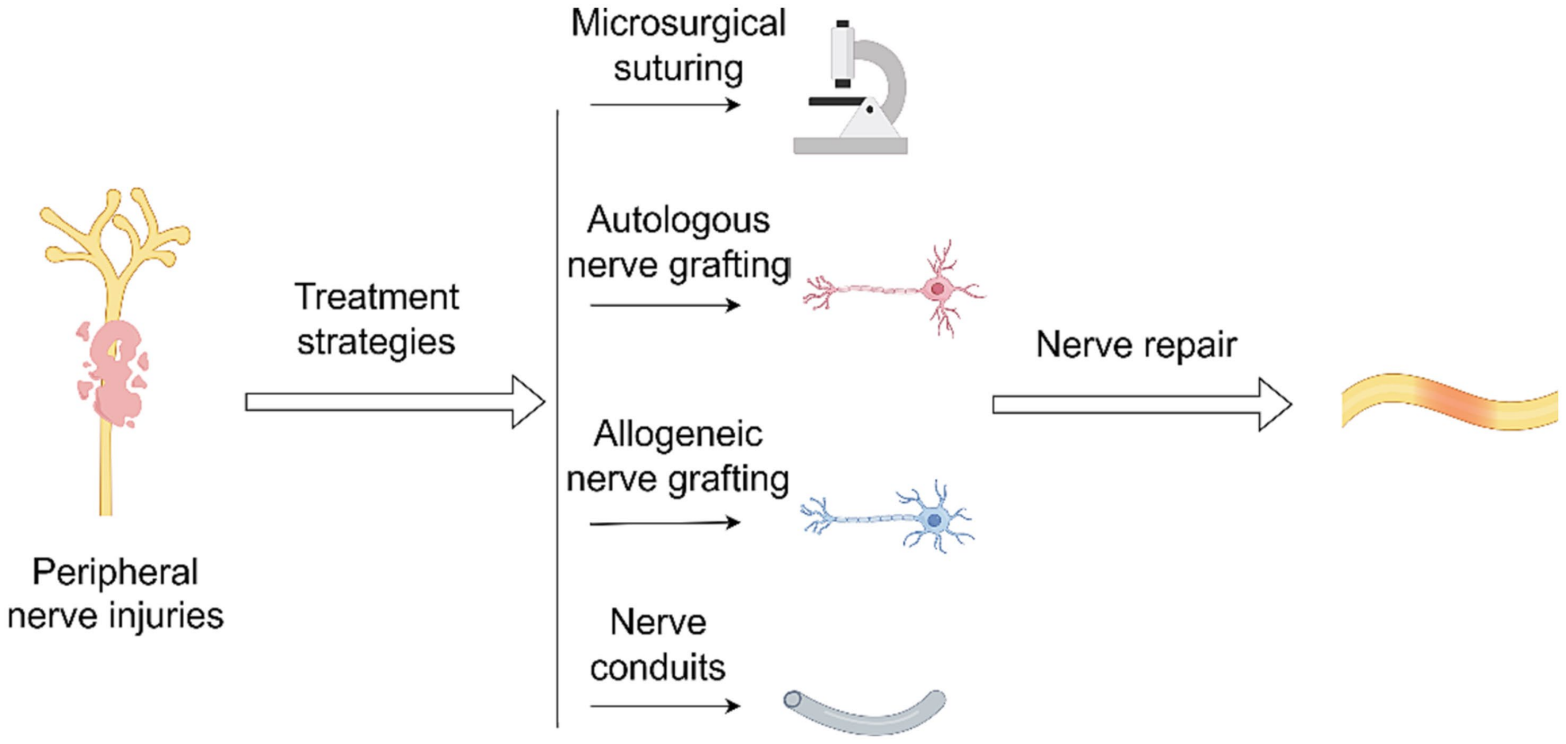
Treatment strategies for peripheral nerve injuries.

To expedite nerve regeneration and span extensive nerve gaps, adjunctive cells, like stem cells or growth factors, have been incorporated into nerve conduits, garnering considerable interest (10). Cell-based nerve conduits exhibit considerable potential in research (11). Nonetheless, their clinical application, both present and prospective, is beset with noteworthy hurdles. Ensuring the safety of cell transplantation stands as one primary challenge. Another significant obstacle is the protracted preparation time for these autologous cell sources, which may risk surpassing the crucial treatment window (12, 13) (Figure 1).

Recent years have witnessed remarkable progress in the field of autologous nerve transplantation. Advances in surgical techniques, nerve graft harvesting, and postoperative care have contributed to improved outcomes and enhanced nerve regeneration. Nursing care plays a pivotal role in the success of autologous nerve transplantation, encompassing preoperative evaluation, meticulous surgical assistance, and comprehensive postoperative management. From monitoring nerve regeneration to implementing tailored rehabilitation plans, nursing care ensures the best possible outcomes for patients undergoing this intricate procedure. This review aims to explore the nuances of nursing care in autologous peripheral nerve transplantation across different anatomical sites. By examining the latest strategies and methodologies, we seek to shed light on how nursing care can optimize postoperative recovery and empower patients to regain functional independence.

## Brachial plexus nerve transplantation care

Brachial plexus nerve transplantation is commonly used for severe upper limb nerve injuries, often resulting from high-energy traumas like car crashes, predominantly affecting young adults. The avulsion of the brachial plexus nerve origins causes significant motor neuron destruction and muscle function impairment (14–16). Traumatic total brachial plexus avulsion (TBPA) presents difficulties as extra plexal nerves must regulate functions for the shoulder, elbow, wrist, and hand. Nerve transfer and free functional gracilis transplantation (FFGT) are current therapies, but complete hand function restoration remains a challenge (17). Recent approaches involve nerve transfer alongside FFGT or double FFGT to enhance hand function (18, 19). Nerve repair often involves suturing the adventitia/intima directly, suitable for acute, localized injuries. Extensive nerve deficiencies may require transplantation, with options like the sural nerve, medial brachial cutaneous nerve, and medial forearm cutaneous nerve. While nerve transplantation avoids donor region cortical remodeling and provides motor bundles, it unavoidably causes donor area injury, and inherent issues like muscle atrophy and neuromuscular dysfunction persist (20–24).

## Preoperative nursing emphasis

Preoperative depression is considered one of the important factors affecting the BPI. The World Health Organization (WHO) reports that more than 17 million Americans (5.9% of the population) suffer from depression (25). Patients experiencing shoulder, hip, and knee replacements have an increased risk of depression when compared to the rest of the population (26–28). A preoperative assessment of depression can be linked with a higher possibility of medical complications (29–31), infection (32), readmission (33), transfusions (34), non-home discharge (35), and length of stay (LOS) for individuals experiencing hip and knee replacement (36, 37). Delays in reconstructive surgery are the primary cause of poor prognosis (38, 39), so the majority of surgeons employ preoperative magnetic resonance imaging (MRI) and neurophysiological tests. Vargas et al. (40) to identify patients with avulsion injuries. Wade et al. (41) mentioned that preoperative (MRI) is used to identify root avulsions, but investigations on its diagnostic efficacy have produced contradictory results. Their survey studies reflected that a minority of diagnosed patients with BPI will not have received a surgical examination for a variety of reasons, including refusal of surgery, hazardous anesthesia, or treatment of other injuries taking precedence. If the percentage of false-negative results is underestimated, this would increase the degree of sensitivity of MRI. Conversely, the diagnostic precision of MRI may be influenced to the downside due to the possibility that patients received treatment based on imaging results rather than the existence of symptoms alone. In addition, they anticipated that the majority of research would be retrospective and some investigations may have obtained a non-representative sample of individuals, which could prejudice the diagnostic precision and raise concerns regarding relevance (41).

### Postoperative nursing emphasis

Efficient pain management post-brachial plexus nerve transplantation is crucial. Nurses assess and address pain intensity, customizing individualized treatment strategies, including opioids, regional anesthesia, and non-pharmacological therapies. It’s essential to ensure correct medication dosages. Regarding splinting techniques, nurses play a vital role in their administration and maintenance, ensuring graft stability and preventing complications (42). Educating patients on three key principles is important:

(a) Examination: check for erythematous indications after removing a splint. Temporary disappearance within 30 min is acceptable; enduring marks require splint adjustment. Engage in joint exercises if joint stiffness occurs.
(b) Washing Instructions: avoid exposing heat-sensitive splints to hot water or heat-emitting sources. Clean splints with cold or lukewarm water and mild soap.
(c) Adjustments: for non-self-constructed splints, avoid attempting modifications. Consult the therapist or center responsible for the splint. Children and adolescents should have orthoses tailored for ongoing growth, necessitating regular follow-up appointments (43).

### Special considerations

In the case of pan-brachial plexus injuries, major functional impairment can result. However, surgical advancements, such as free-functioning muscle transfers, have led to significant improvements in functional ability. However, individuals with brachial plexus injuries still face challenges post-surgery, including persistent pain, occupational changes, appearance concerns, dependence on others, and difficulties with daily tasks (44). To enhance healthcare services for BPI patients, several considerations should be followed post-transplantation surgeries:

(A) Psychological support: BPI patients may experience diverse emotional responses, including worry and dissatisfaction. Nurses play a crucial role in offering emotional assistance and facilitating counseling services as needed.
(B) Family involvement and education: Family engagement is vital for patient rehabilitation. Nurses provide families with education on postoperative care, including wound care, splint management, and prescribed exercises.
(C) Occupational therapy: This plays an essential role in helping patients regain independence in daily activities. Nurses collaborate with occupational professionals to ensure a smooth transition from rehabilitation to occupational therapy (45).

Brito et al. (46) examined the experiences of individuals who received free-functioning muscle transfers for Pan-BPI. Despite access to extensive medical resources, patients faced challenges related to physical impairments, changes in relationships, reliance on pain medication, self-concept adjustments, and resuming important roles like employment. Providers must address personal and social needs, including pain management, depression, adapting to a new self-identity, and re-engaging in life roles. The Continuity of healthcare professionals is crucial for effective therapy. Therefore, a broader application of continuity of care for BPI individuals and relevant community support initiatives is essential. Strategies should be explored to improve care for patients in areas with limited access to specialized BPI treatment. Occupational health practitioners are well-positioned to provide services for adaptation, pain management, psychological effects, and societal reintegration. This study provides valuable insights for healthcare practitioners, planners, and funders supporting BPI individuals (46).

### Donor nerve requirements

There are some vital requirements for donor nerve transplantation in BPI surgery as follows: (i) the selection of a suitable and compatible donor nerve is a crucial factor in the transplantation of brachial plexus nerves. Ideally, the selection of the donor nerve should align with the specific type of nerve fibers needed for the recipient’s place (it will be for sensory or motor purposes). (ii) The factor of proximity has played an essential part in minimizing graft length and tension, hence influencing the success of the transplant. It is better to have a nerve in close proximity to the site of injury. (iii) Functional significance at donor location: the selection of a donor nerve should prioritize minimizing the functional consequences associated with its loss at the donor site. Generally, sensory nerves in areas of less significance are favored. (iv) Surgical access: the practical aspects of accessibility and convenience of harvesting the donor nerve are important factors to be taken into account. Surgeons must possess the capability to efficiently and effectively obtain and extract the donor nerve while causing minimal disturbance (47, 48).

A nerve transfer for elbow extension is recommended in cases where grafting is not possible, such as root avulsions. A variety of nerves are used in these transfers, including the suprascapular, phrenic, contralateral C7 root, partial medial or ulnar, spinal accessory, or intercostal nerves. Restoration of elbow flexion is the first priority, followed by shoulder external rotation and grasp function. Elbow extension is considered less often but becomes relevant when using a functional free muscle transfer that crosses the elbow to enhance grasp capabilities (49). In Nagano (50) documented the use of multiple nerve transfers for lesions specifically affecting the C5-6 region, including the utilization of intercostal nerves to reinnervate the musculocutaneous nerve.

## Upper limb nerve transplantation care

Upper limb nerve transplantation is a highly specialized intervention designed to restore both sensory and motor capabilities in the hands and arms. Its main goal is to address the impaired finger movement resulting from nerve damage or loss. Nerve transfer presents a viable approach for the restoration of function, with the primary objective of revitalizing the paralyzed muscles in the upper limbs (51). The basic justification for implementing nerve transfer procedures in individuals with tetraplegia is the redirection of intact nerve axons originating proximal to the site of injury toward paralyzed muscle-nerve cells located below the site of injury. This process effectively circumvents the damaged region of the spinal cord. Axons that are in healthy condition and subject to voluntary control have regenerative properties, wherein they undergo a process of restoration by extending from the donor’s nerves. This regenerative phenomenon aims to reinstate the ability to control muscles that were previously rendered immobile due to a spinal cord injury (SCI) (52).

**Figure 2:**
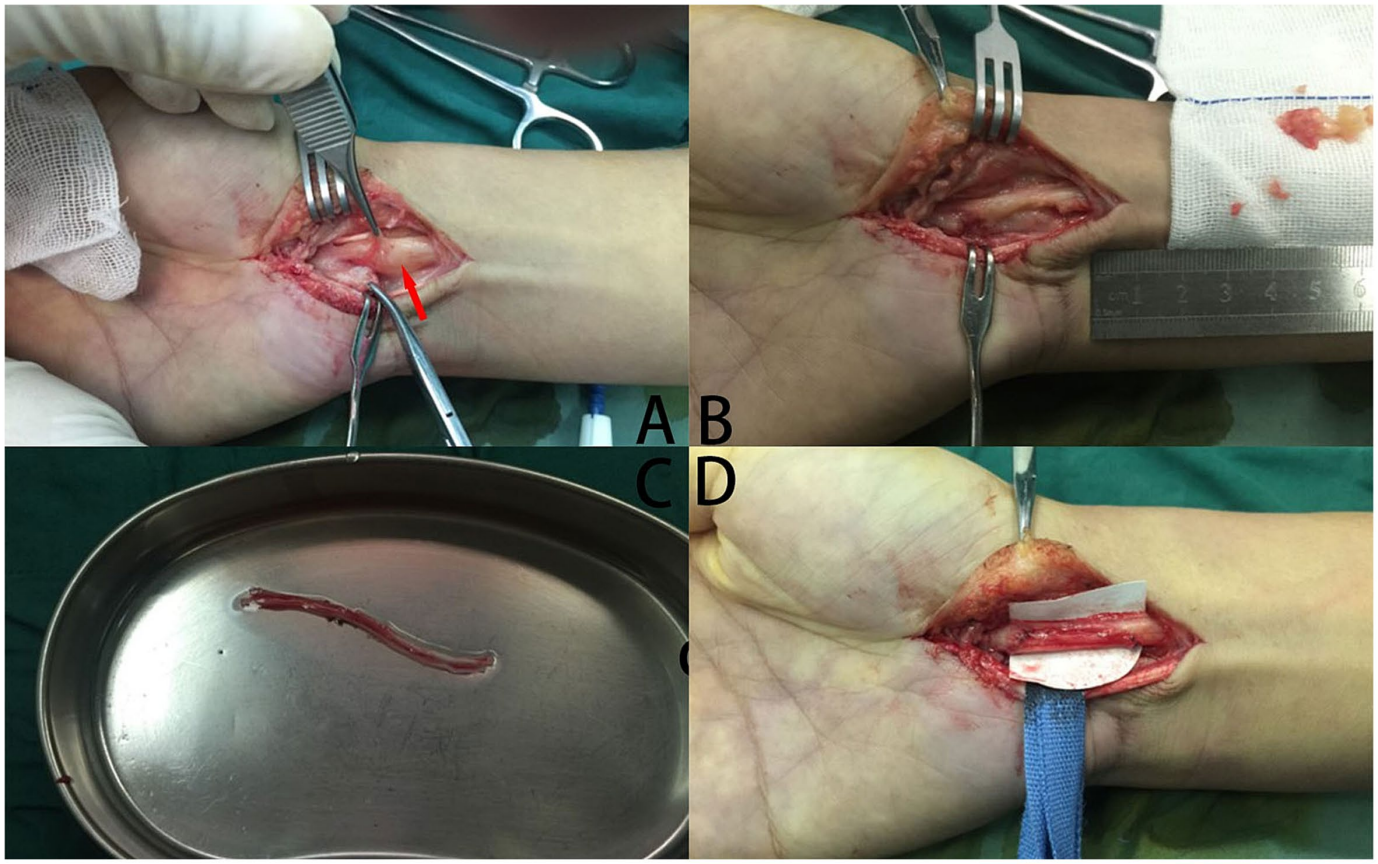
The procedure of tumor resection in the median nerve and a sural nerve graft. **(A)** A tumor (red arrowhead) grew in the median nerve, which was encased by the nerve tissue. **(B)** The tumor was excised, which causes the median nerve defects. **(C)** The sural nerve has been incised. **(D)** Postoperative images of autologous nerve transplantation.

**Figure 3:**
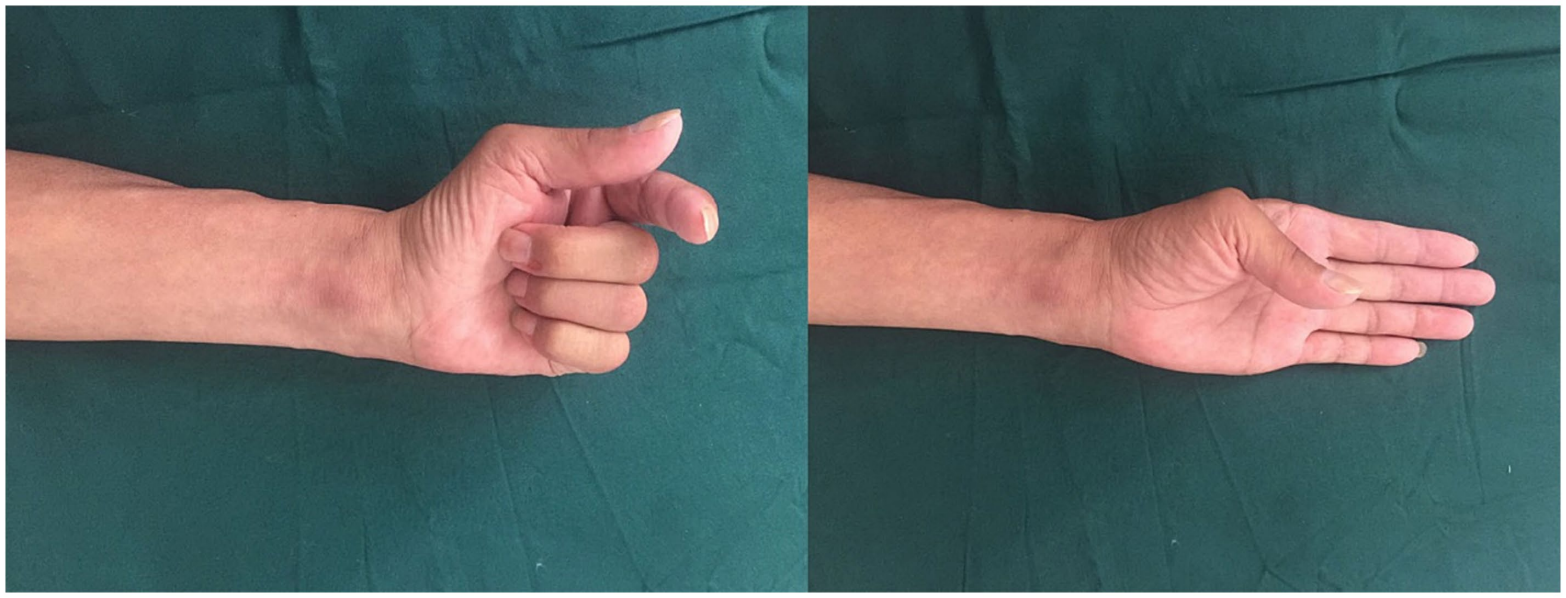
Postoperative functional assessment at 6 months.

In our case, a 41-year-old male complained of numbness in the left thumb, index finger, and middle finger and was characterized by abnormal palmar opposition of the thumb. His motor nerve conduction and sensory nerve conduction studies indicated a lesion of the median nerve. After administering general anesthesia, the patient underwent tumor resection in the median nerve and received a sural nerve graft. After 1 month, the sensation in the fingertips had recovered. The function of palmar opposition of the thumb returned to completely normal within 6 months (Figures 2, 3).

### Preoperative nursing emphasis

The successful outcome of upper limb transplantation is contingent upon both the meticulous care provided to the patient post-operation and the efficacy of the treatment itself. In order to preserve the viability of the transplanted part, it is imperative to implement a comprehensive regimen consisting of vigilant pharmacological therapy, intensive physical therapy, essential psychological support, and regular testing. Certain medicines are required to be administered continuously over the lifespan of the graft (53). In order to adequately prepare for the postoperative care of patients receiving hand transplantation, it is essential to establish a select group of nurses who possess clinical knowledge in the specialized care of hand and microsurgery individuals. In addition, these nurses must receive specialized educational training pertaining to the provision of care for patients enduring transplantation procedures. The patient is admitted to the designated medical facility prior to the surgical procedure and assigned to a room equipped with a high-efficiency particle air filter.

In order to foster a sense of security and contentment, it is recommended that the nurse greets the patient and their family in a kind, engaged, caring, and non-judgmental manner (54, 55). An unhurried attitude should characterize this approach. The patient and their family are provided with a comprehensive orientation to the unit and call framework, with an emphasis on promoting open communication with the nursing staff and physicians. Nurses conduct an initial evaluation of the patient and their family, including a comprehensive psychosocial assessment. In anticipation of the surgical procedure, hand transplant preoperative requirements are recorded and completed (56, 57).

### Postoperative nursing emphasis

Nurses conduct a comprehensive physical examination of the recipient after the recipient is transferred from the postoperative care unit to the hospital ward. Furthermore, the circulation condition of the transplant is evaluated on an hourly schedule. During transplant surgery and afterwards, a pulse oximeter is used to monitor the unaffected and transplanted limbs’ blood flow. Using a device for measuring skin temperature, the surface of the transplanted hand’s fingers is compared with those on the unaffected limb (58). Temperature measurements should be recorded at a minimum of 30°C or above. It is imperative to promptly notify the attending physician immediately of a decrease in body temperature or alteration in the circulatory condition. Keeping the recipient’s room at 24°C is recommended. The consumption of caffeine is contraindicated for the patient, and smoking is strictly restricted. The family is advised to refrain from smoking before their visits to the beneficiary. Nurses evaluate pain management, nutritional status, physical activity level, bowel and bladder function, and anti-embolic measures regularly following surgery (54). A specific pharmaceutical regimen facilitates immunosuppression, after which the transplant physician and transplant coordinator monitor the progress. The coordinator conducts regular trips to the recipient, providing education to both the recipient and their family on topics such as medications, activity levels, environmental considerations, and other relevant variables (59, 60). Due to the recipient’s immunosuppressed condition, it is imperative for the nurse responsible for their care to exert utmost effort in coordinating visits from family members and pertinent personnel, with the aim of reducing the frequency of individuals entering the room. The discharge nurse is responsible for providing a comprehensive explanation of surgical discharge guidelines, including (1) the time of administration for all medications, including information on pharmaceutical types, dosages, and the corresponding profiles of adverse effects. (2) Timetable for mandatory laboratory examinations. (3) The scheduling of physician follow-up visits. (4) Wound care. (5) The evaluation of hand color, warmth, and capillary refill. (6) The surveillance of blood pressure and temperature. (7) The importance of hygiene and the prevention of opportunistic infections (61, 62).

### Special considerations

Effective communication between the hand transplantation hospital and the hand therapist and/or orthotist involved in the recipient’s care is crucial. Physical and occupational therapists should actively view real-time video recordings of therapy to understand potential variances in tendon architecture and surgical intricacies affecting rehabilitation (27). Additional electrodes can be placed along the median and ulnar nerves above the transplant site after surgery. Transcutaneous electrical nerve stimulators can be used when needed, either in the recovery room or at the initial dressing change. The recipient should undergo daily hand rehabilitation for at least 3 months, followed by ongoing care from a local hand therapist (63, 64). Using a dynamic crane outrigger splint and an extension block at the metacarpophalangeal joint can significantly improve functional outcomes, similar to hand replantation (65, 66). Exercise programs are based on robust tendon weave repair techniques. Scar care, including compression materials, can start around 4 weeks post-transplantation. Hand-based anti-claw splints may be used intermittently after 3 weeks. For functional assessment, Carroll’s test and the Disability of the Arm, Shoulder, and Hand (DASH) questionnaire are used (67). Immunosuppressive treatments for limb transplantation include drugs for induction and maintenance. Induction phase drugs typically include a calcineurin inhibitor, an antimetabolite, a monoclonal antibody, and steroids. Maintenance therapy involves lower doses of steroids, antimetabolites, and calcineurin inhibitors. Steroid-free maintenance therapy has shown promising results (68–70). The hospital should maintain an adequate supply of antibiotics, antihistamines, opioids, gastrointestinal prophylaxis medications, and laxatives for surgery. Anticoagulation requires aspirin, low molecular weight dextran, and heparin. Local anesthetic drugs like bupivacaine and ropivacaine, with epinephrine, should be available for potential nerve blocks (54).

### Donor nerve requirements

To achieve optimal results in sensory and motor restoration for the recipient’s hand, several key factors must be considered in selecting donor nerves. Compatibility with the specific type of fibers needed is crucial for successful integration and functional recovery. Proximity to the recipient site is also essential to minimize graft length and the stress associated with it. Choosing nerves close to the damaged site increases the chances of successful transplantation (71). Careful selection of donor nerves is imperative to minimize adverse effects on the functionality of the donor location. It’s generally preferable to choose sensory nerves in less vital regions to reduce negative impacts on the donor site (72, 73). A study by Javeed et al. (51) reported that upper extremity function could be restored using single, double, or triple nerve grafts. Various nerve transfers were performed based on injury severity, remaining motor function, and electrodiagnostic signs of motor neuron damage. The Medical Research Council graded donor nerves 4–5 on their clinical motor strength. In the context of nerve transfer pairings, the supinator branch of the radial nerve was combined with the posterior interosseus nerve to facilitate hand opening, finger, and wrist extensions. Similarly, the brachialis branch of the musculocutaneous nerve was paired with the anterior interosseus nerve fascicle of the median nerve to enable pinch and finger flexion. Additionally, the spinal accessory nerve was linked with the posterior deltoid motor branch of the axillary nerve to support elbow extension (51).

## Lower limb nerve transplantation

Patients with severe trauma may experience discomfort and functional impairment due to PNI. A prevalence rate of 1.2% has been observed in lower extremity injury patients developing PNI, which increases the risk of chronic pain and the need for therapy (74). In the past, upper extremity peripheral nerve reconstruction has received more attention than lower extremity peripheral nerve reconstruction. Nerve transfers in the lower extremities pose challenges due to longer nerve lengths, potentially leading to unfavorable outcomes (75). Lower limb nerve transplantation aims to restore sensory perception and motor control. Sensory deficiencies can affect proprioception and balance, while motor impairments can reduce muscle strength and alter gait (76). Treatment for lower extremity nerve injuries varies based on severity and nerve gap length. Nerve conduits are utilized for nerve gaps measuring less than three centimeters, while direct healing is favored for smaller gaps. Autografts or allografts are employed for gaps exceeding 3 cm; nevertheless, their utilization may be limited due to factors such as scar tissue, hemostasis, and infection (77).

Individualized approaches considering the advantages and limitations of each therapeutic intervention are essential for managing lower extremity nerve injuries.

### Preoperative nursing emphasis

A variety of essential nursing interventions characterize the preoperative period of lower limb nerve transplantation. The purpose of these treatments is to enhance patients’ empowerment via the acquisition of knowledge, provision of emotional support, and the facilitation of physical preparedness for surgical procedures. The primary focus throughout this stage is on comprehensive care, acknowledging the interconnectedness of the patient’s physical and mental health. For example, in the context of patients with fractures, normal nursing care mostly centers on the observation of their health and the provision of general recovery instructions, sometimes falling short of meeting customized needs. Fast-track surgery (FTS) aims to expedite the rate of postoperative recovery, reduce physiological and psychological stress (78), and facilitate early discharge from the hospital for patients (79). This is achieved by the use of various optimal perioperative nursing interventions, such as less invasive surgical procedures.

The perioperative phase encompasses several key components, such as pre-hospitalization education, preoperative fasting guidelines, postoperative vital sign monitoring, and the provision of analgesics as needed. Participants were permitted to consume water and food once the gas had gone through the anus, to have the catheter removed when spontaneous urination had been regained, and to engage in recovery activities at their discretion. The nursing intervention, as described by Chen et al. (80), includes comprehensive patient education using various methods such as verbal, visual, and written materials covering topics like hospital procedures, surgery concepts, pain management, fracture diagnosis, and healing processes. Nutritional guidance involves reducing preoperative food and water restrictions, closely monitoring vital signs post-anesthesia, and gradually introducing meals. Pain management is tailored to the patient’s pain severity, using the visual analog scale (VAS) for assessment and administering appropriate analgesics. Additionally, patients receive guidelines for functional exercises, including passive and active routines post-surgery, ensuring comprehensive care and support.

### Postoperative nursing emphasis

The nurses engaged in effective communication with patients who expressed reluctance to participate in post-surgical recovery exercises owing to intense pain. They employed a patient and empathetic approach, elucidating the significance of early engagement in functional exercises for rehabilitation. The researchers implemented suitable methodologies to alleviate the discomfort experienced by the patients, followed by guiding and engaging in functional exercises. Following the dissipation of the anesthetic, the leg in concern was raised at an angle of 15° to facilitate the systematic massaging of the toes and pulps. Patients performed toe flexion and extension exercises for 10 s, as directed by the nursing staff, followed by a 5-min rest period. Post-operative exercises consisted of ankle pump exercises, relaxation, and a contraction of the quadriceps femoris on the first day, contraction of the gluteus maximus on the second day, calf muscle contraction and exercise of the back muscles on the third day, straight leg raising on the fourth day, knee joint bending and extending on the fifth day, and hip joint movements on the sixth day (80). Postoperative care includes the following key aspects:

Swelling Assessment: Swelling in the affected limb was evaluated before surgery and at 1, 2, 3, and 4 weeks post-surgery, categorized into three levels: Level I (modest swelling), Level II (evident swelling with increased skin temperature), and Level III (brilliant swelling with tension blisters).

Pain Evaluation: Pain intensity was measured using the VAS scale before surgery, immediately after surgery, and at 6, 24, and 72 h post-surgery, with higher scores indicating more severe pain.

Complications Monitoring: A 6-month follow-up assessed the occurrence rates of constipation, urinary tract infection (UTI), lung infection, and lower limb deep vein thrombosis. Deep vein thrombosis diagnosis used color Doppler ultrasonography, checking for blood flow absence and sound. Lower Extremities Function: Kostuj et al. (81) assessed ankle and knee joint function at 3 and 6 months post-surgery using the AOFAS Ankle Hindfoot Scale and Lysholm criteria. The AOFAS scale comprises nine components, each with a maximum score of 100 points, evaluating pain levels, functional abilities, independent movements, limitations in everyday activities, gait patterns, forefoot and backfoot activities, ankle and heel stability, and foot alignment. Scores range from ≤50 (poor) to 90–100 (exceptional). The Lysholm criteria cover factors like discomfort, swelling, crouching, encouragement, instability, limping, and interlocking, with scores >84 (normal), 66–84 (acceptable), and <65 (poor).

### Special considerations

Both amputation and limb salvage therapies for lower limb injuries have limitations. Functional and psychological outcomes often remain unsatisfactory compared to the patient’s initial condition 2 years post-injury (82). Despite uncertainties regarding the applicability of upper extremity allotransplantation outcomes to lower extremity cases due to various factors, there is hope for lower extremity allotransplantation based on an examination of upper extremity transplantation advantages and disadvantages (83). Potential outcomes for lower extremity allotransplantation encompass several factors. First, lower limb anatomy is simpler, with fewer intricate structures, reducing operative complexity and anesthesia time, likely resulting in fewer complications (83). Second, intrinsic muscle reinnervation’s significance is lower in the lower limbs compared to the upper limbs. The restoration of proximal thigh muscle function, essential for ambulation, is prioritized over intrinsic reinnervation, making lower extremity allotransplantation potentially beneficial for converting above-knee to below-knee amputations (84). Third, while concerns about immunosuppression exist, lower extremity allotransplants, especially above the knee, may contain hematopoietically active bone marrow, potentially reducing the need for immunosuppressive medications (85, 86). Finally, lower limb allotransplantation is expected to significantly improve recipients’ quality of life by restoring sensation, function, and integrity, similar to the benefits observed in upper extremity transplant recipients. These improvements may also enhance body image, autonomy, and social reintegration (87, 88).

### Donor nerve requirements

Primary repair is recommended when the nerve damage is complete, without significant gaps, and can be reconnected without tension. Autogenous nerve grafts like the sural nerve are ideal when the nerve cannot be repaired without tension. This method requires intact proximal and distal nerve targets and a donor nerve with expendable functionality (89). Nerve conduits can guide nerve regeneration but are limited to shorter segment abnormalities. When direct repair or grafting is not feasible, nerve transfers, connecting a functional donor nerve to a non-functional recipient nerve, can be used. Nerve transfers have gained attention in lower extremity PNI due to the long distances involved, potentially avoiding the zone of injury. For common peroneal nerve injuries, neurolysis restored useful function in about 88% of cases. Direct repair achieved similar results in 84% of patients. However, the recovery probability decreased with longer grafts (90). Nerve transfer is recommended in cases meeting specific criteria: no motor function between 3 and 12 months post-injury, too great a gap for primary repair or grafting, and unavailability of the proximal nerve end for repair. Rehabilitation aims to establish a stable hip joint, normal walking patterns, and a protective feeling in the plantar region of the foot (91). Nerve transfers in the lower extremities are used to provide protective sensation to the foot’s plantar side and address painful neuropathies and neuromas. Nerve transfers are not preferred when other surgical methods might yield similar results with less morbidity. Motor nerve transfers aren’t recommended if there’s a delay of more than 12–18 months since the injury or if donor nerve strength is below BMRC grade M4 (92). Transferring motor nerves requires direct end-to-end coaptation, verifying no muscle contraction in the recipient nerve. In sensory nerve transfers, either end-to-end or end-to-side transfers can be used effectively. Sensory nerve transfers aim to regain essential sensations using a donor nerve lacking vital sensations (93–95). Nerve transfers from the proximal motor branches of the tibial nerve to common or deep peroneal nerve injuries have shown promise. In one study, seven out of nine pediatric patients with common peroneal nerve palsy achieved at least M4 rehabilitation in ankle dorsiflexion over 6 months after the transfer (96). Overall, primary repair, grafts, conduits, and nerve transfers are valuable tools in addressing peripheral nerve injuries, each with its own indications and considerations (Table 1).

**Table 1:** Nursing care summary for nerve transplantation.

Injured area	Preoperative nursing	Postoperative nursing
Brachial plexus injury	Assess for depression Educate on surgery process MRI and tests to ID avulsion injuries	Pain management Splinting education Psychosocial support
Upper limb nerve damage	Specialized nurse training Patient/family education Comprehensive assessments	Frequent graft monitoring Immunosuppression management Rehab and testing
Lower limb nerve injury	Patient education Nutrition guidance Pre-op preparation	Swelling and pain checks Functional exercises Complication monitoring

## Application of new care approaches

### Personalized rehabilitation plans

The primary objectives of Rehabilitation Medicine are to enhance a patient’s overall quality of life, physical and psychological functioning, especially in the context of disabilities or illness. Rehabilitation Medicine adopts a comprehensive approach known as the biopsychosocial interdisciplinary and multimodal approach (97).

Rehabilitation after hand and upper limb transplantation, which is the focus here, involves a multi-stage process:

(i) Early Stage (0–6 weeks): The primary goal is to educate patients about safeguarding and adopting the right posture for the insensate limb. A thermoplastic volar resting splint is used to protect bone fixation and soft tissues. It’s worn for 6 weeks, except during physical activity and certain maintenance tasks. Edema is managed using circumferential measures, posture, massage, mobilization, and compression. Active and passive movements are initiated between three and 5 days post-transplantation to prevent joint stiffness and tendon adhesions. Sensory-motor re-education is encouraged through the prehabilitation motor imagery plan.
(ii) Intermediate Stage (6–12 weeks): Motor and sensory restoration are closely monitored, and necessary adjustments to splinting and exercises are made. The volar resting splint is used mainly at night, while daytime splints facilitate functional tasks. Motor relearning is incorporated, with a focus on achieving a complete passive motion range within 12 weeks. Strengthening exercises are introduced, and massage and compression are used for scar and edema management.
(iii) Late Stage (12 weeks and more): Outpatient visits are gradually reduced based on individual needs. Patients are encouraged to maintain their home exercise routine. The treatment evolves to enhance muscular strength, endurance, and overall functional autonomy. Sensory re-education continues, including static and dynamic localization, texture and form discrimination, immersion, and stereognosis. Patients are advised to integrate sensory re-education into their daily routines and experience various sensory stimuli. Rehabilitation focuses on identifying and pursuing long-term therapeutic goals (98).

This rehabilitation process is tailored to patients undergoing hand and upper limb transplantation, considering factors such as the level of transplantation and individual health conditions.

### Integration of advanced techniques

Peripheral nerve regeneration after injury is challenging, especially for proximal nerve injuries where axons must cover long distances at a slow rate of 1 mm/day (99).

Nerves severed in experimental injuries may regenerate if repaired within 3 months (100). Delaying repair for four to 6 months significantly reduces regeneration capacity to only 33% of normal levels (99). Despite progress in peripheral nerve restoration, satisfactory clinical outcomes remain elusive, often resulting in long-term sensorimotor impairment and neuropathic pain in patients (101, 102). An effective approach to expedite peripheral nerve regeneration is electrical stimulation (ES) applied directly to the injured nerve. ES enhances early regeneration processes, promoting neuronal survival and axonal sprouting (103). In rodent injury models, ES has shown potential in enhancing neuron regeneration in various nerve injuries (104–106). *In vitro* studies suggest that ES increases intraneuronal cyclic adenosine monophosphate (cAMP) levels and nerve growth factor (NGF) levels, aiding regeneration (107). The specific cellular mechanisms of ES in axonal regeneration are not fully understood. ES mimics normal calcium influx after nerve damage, providing a retrograde signal that activates regeneration-promoting processes (108, 109). ES accelerates axonal growth and upregulates regeneration-related genes (105, 110, 111). ES also influences cAMP, linked to neurite outgrowth and axonal guidance (112, 113). ES triggers cAMP production, activating pathways promoting brain-derived neurotrophic factor (BDNF) and neurite development (114). BDNF inhibits cAMP breakdown, maintaining elevated levels (109, 115). Recent research explores ES’s impact on downstream pathways, including PTEN downregulation, a growth inhibitor (116). PTEN inhibits the PI3-K/Akt pathway crucial for regeneration (117, 118). Inhibiting PTEN facilitates peripheral nerve regeneration (119), suggesting ES promotes the PI3-K/Akt pathway (116). Additionally, using growth factors (GFs) to facilitate nerve regeneration is explored. GFs activate signaling cascades, but their short effectiveness and quick inactivation are limitations. Nerve conduits with controlled GF release support axonal regeneration and functional recovery (120).

## Conclusion

Autologous peripheral nerve transplantation care is experiencing substantial progress across various anatomical sites. Through the adoption of personalized rehabilitation plans, advanced techniques, and effective complication management, enhanced postoperative recovery can be achieved, providing patients with clearer pathways toward functional restoration.

## Author contributions

GX: Writing – original draft. XZ: Writing – review & editing. YD: Writing – review & editing. AA: Writing – review & editing. HZ: Writing – review & editing. SE: Writing – review & editing. VK: Writing – review & editing. MA: Writing – review & editing. OA: Writing – review & editing. SA: Writing – review & editing. HL: Writing – review & editing.
