# Corrigendum: Advancements in autologous peripheral nerve transplantation care: a review of strategies and practices to facilitate recovery

**DOI:** 10.3389/fneur.2025.1590996

**Published:** 2025-05-12

**Authors:** Guoying Xu, Xiaodi Zou, Yanzhao Dong, Ahmad Alhaskawi, Haiying Zhou, Sohaib Hasan Abdullah Ezzi, Vishnu Goutham Kota, Mohamed Hasan Abdulla Hasan Abdulla, Olga Alenikova, Sahar Ahmed Abdalbary, Hui Lu

**Affiliations:** ^1^Operating Theater, Shaoxing City Keqiao District Hospital of Traditional Chinese Medicine, Shaoxing, Zhejiang, China; ^2^Department of Orthopedics, The Second Affiliated Hospital of Zhejiang Chinese Medical University, Hangzhou, China; ^3^Department of Orthopedics, The First Affiliated Hospital, Zhejiang University, Hangzhou, Zhejiang, China; ^4^Department of Orthopedics, Third Xiangya Hospital, Central South University, Changsha, Hunan, China; ^5^School of Medicine, Zhejiang University, Hangzhou, Zhejiang, China; ^6^Department of Neurology, Republican Research and Clinical Center of Neurology and Neurosurgery, Minsk, Belarus; ^7^Department of Orthopedic Physical Therapy, Faculty of Physical Therapy, Nahda University in Beni Suef, Beni Suef, Egypt

**Keywords:** peripheral nerve injuries, autologous nerve transplantation, nursing care, functional impairments, rehabilitation

In the published article, there was an error in affiliation 4. Instead of “Faculty of Medicine, School of Biomedical Sciences, The Chinese University of Hong Kong, Shatin, Hong Kong SAR, China”, it should be “Department of Orthopedics, Third Xiangya Hospital, Central South University, Changsha, Hunan, China”.

The authors apologize for this error and state that this does not change the scientific conclusions of the article in any way. The original article has been updated.

